# Litter Detection with Deep Learning: A Comparative Study

**DOI:** 10.3390/s22020548

**Published:** 2022-01-11

**Authors:** Manuel Córdova, Allan Pinto, Christina Carrozzo Hellevik, Saleh Abdel-Afou Alaliyat, Ibrahim A. Hameed, Helio Pedrini, Ricardo da S. Torres

**Affiliations:** 1 Institute of Computing, University of Campinas, Avenue Albert Einstein, Campinas 13083-852, Brazil; manuel.cordova@ic.unicamp.br (M.C.); helio@ic.unicamp.br (H.P.); 2 Brazilian Center for Research in Energy and Materials (CNPEM), Brazilian Synchrotron Light Laboratory (LNLS), Campinas 13083-100, Brazil; allan.pinto@lnls.br; 3 Department of International Business, NTNU—Norwegian University of Science and Technology, Larsgårdsvegen 2, 6009 Alesund, Norway; christina.hellevik@ntnu.no; 4 Department of ICT and Natural Sciences, NTNU—Norwegian University of Science and Technology, Larsgårdsvegen 2, 6009 Alesund, Norway; alaliyat.a.saleh@ntnu.no (S.A.-A.A.); ibib@ntnu.no (I.A.H.); 5 Farm Technology Group and Wageningen Data Competence Center, Wageningen University and Research, 6708 PB Wageningen, The Netherlands

**Keywords:** litter, marine litter, citizen science, litter detection, object detection, neural networks, deep learning, machine learning, portable devices

## Abstract

Pollution in the form of litter in the natural environment is one of the great challenges of our times. Automated litter detection can help assess waste occurrences in the environment. Different machine learning solutions have been explored to develop litter detection tools, thereby supporting research, citizen science, and volunteer clean-up initiatives. However, to the best of our knowledge, no work has investigated the performance of state-of-the-art deep learning object detection approaches in the context of litter detection. In particular, no studies have focused on the assessment of those methods aiming their use in devices with low processing capabilities, e.g., mobile phones, typically employed in citizen science activities. In this paper, we fill this literature gap. We performed a comparative study involving state-of-the-art CNN architectures (e.g., Faster RCNN, Mask-RCNN, EfficientDet, RetinaNet and YOLO-v5), two litter image datasets and a smartphone. We also introduce a new dataset for litter detection, named PlastOPol, composed of 2418 images and 5300 annotations. The experimental results demonstrate that object detectors based on the YOLO family are promising for the construction of litter detection solutions, with superior performance in terms of detection accuracy, processing time, and memory footprint.

## 1. Introduction

Nowadays, litter is one of the greatest challenges [1], due to its ubiquity, nature and scale, with dire consequences not only for freshwater and marine ecosystems [2,3,4,5], but also, arguably, for urban environments and human health. For example, plastic items can cause lethal damage to aquatic and terrestrial species through entanglement, gut perforation, and starvation [6]. Moreover, plastic decomposes, creates microplastics and, ultimately, nanoplastics which easily enter the food chain [7].

Additionally, one of the main concerns is the accumulation of litter not only in the urban area [8], but, mainly, in ecological areas [9,10]. To investigate and tackle this challenge, decision-makers require reliable data—not only on the sources of waste, but also on its composition, distribution and magnitude over large geographic areas. One way of providing technological support is to automate the process of litter logging and litter detection, especially in areas of difficult access, such as forest and mountains, so as to make the process more effective for volunteers and researchers and enable the processing of large datasets, while minimizing resources, fatigue, and even risks.

During the last years, some research initiatives have investigated suitable computational approaches to support surveys on waste and litter occurrence and distribution. Several works are focused on the Internet of Things (IoT), where sensors are used to monitor city cleanliness [11,12,13]. Researchers have also focused efforts on image-based approaches for marine debris, plastic and even microplastic identification [14,15,16,17,18,19]. However, image acquisition procedures rely on microscopes, webcams, and even aerial surveys, which are costly and often unavailable to volunteers and citizen scientists. Detection and recognition often explore color and shape information, combined with threshold-based classification methods, costly and time-consuming visual inspection. However, none of these alternatives consider the use of mobile-based image acquisition procedures. Moreover, the use of deep learning approaches, especially adapted for devices with constrained processing capabilities, such as mobile phones, has not yet been investigated, a gap that still needs to be filled.

Currently, most of the state-of-the-art solutions for object detection and recognition exploit CNN approaches [20], which rely on the use of deep architectures, such as VGG [21]. Despite their success, such solutions are computationally costly in terms of processing, as well as memory and storage footprints. Therefore, their use is unfeasible in practice, in several applications with computational constraints, such as mobile devices. A suitable alternative explored in the literature refers to the use of “mobile” CNN architectures [22,23,24], i.e., lightweight CNN architectures specifically designed for mobile devices. However, to the best of our knowledge, there is currently no study concerning the assessment of suitable litter detectors using neural network architectures, especially in scenarios involving devices with low-processing capabilities.

Moreover, despite the numerous cloud computing services for machine learning, its response time is not enough for real-time applications, especially in natural settings, such as beaches and forests, among others, impacted by latency fluctuation or even without network coverage. Moreover, data transfer over the network involves more energy consumption [25,26]. In this vein, efficiency, local processing and lower energy consumption are some of the advantages of using lightweight neural networks [25,27].

**Figure 1:**
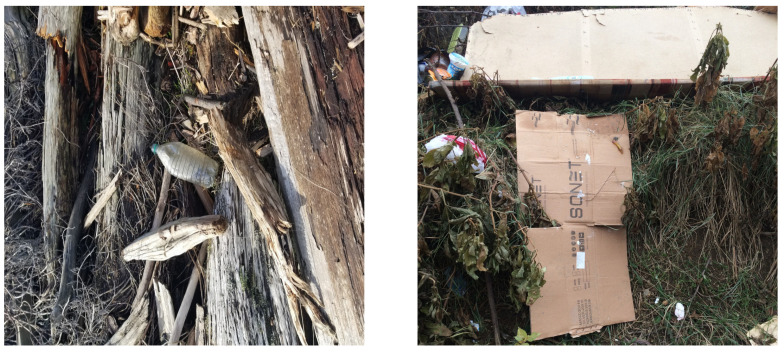
Litter detection in real scenarios.

We performed a comparative study in the context of litter and waste detection using well-known state-of-the-art CNN architectures (e.g., Faster RCNN [28], Mask-RCNN [29], EfficientDet [30], RetinaNet [31] and YOLO-v5 [32]) and lightweight neural networks approaches (e.g., EfficientDet-d0 [30] and YOLO-v5s [32]). Those approaches were compared not only in terms of their effectiveness (detection quality), but also in terms of their efficiency, i.e., detection time and storage requirements. Additionally, the identification of effective machine learning approaches to confront the issue of litter demands the use of comprehensive datasets, i.e., the set of images used to train the models impacts the performance of the methods when tested in real-world settings. Detecting litter in such scenarios is a very challenging problem. Figure 1 provides examples where complex natural backgrounds and the presence of different kinds of litter complicate the detection task. Currently, there exist some litter datasets; however, most of them were built in controlled setups, i.e., with only one instance of litter per image [33,34] or taken in indoor scenarios for recycling [35,36]. Approaches developed based on such image collections cannot be generalized for real-world scenarios. To the best of our knowledge, TACO [37] is the only publicly available dataset containing 1500 images from realistic outdoor scenario. For this reason, herein, we present a new dataset, named “PlastOPol,” which is based on images taken through the Marine Debris Tracker with the goal of giving, to the computer science and environmental communities, a new set of 2418 images with the presence of litter in a realistic context covering several types of environments, i.e., urban, beaches, forests and flint fields, and including different types of litter, including plastic, glass, metal, paper, cloth and rubber, among others.

In summary, this paper aims to fill the following gaps in the literature: assessing the effectiveness of lightweight neural networks in detecting litter in real-world settings and crowded image backgrounds and their ability to run in mobile devices with memory constraints (henceforth referred to as efficiency). The ultimate aim is to aid the scientific community in developing simple, cost-effective tools to automate part of the work conducted by volunteers and citizen scientists when collecting and recording litter in the environment, to make more data available to science and ultimately inform targeted policies and management measures. Our contributions are twofold, as follows:

(1) Comparative study of state-of-the-art deep learning solutions to support image-based litter and waste detection;
(2) Introduction of a new dataset, PlastOPol, composed of 2418 images in real-world settings with 5300 litter annotations.

The experiments considered two datasets, namely, PlastOPol and TACO [37], and aimed to assess the performance of the detectors using mobile devices. The experimental results showed that YOLO-v5x [32] outperformed the other state-of-the-art methods in both datasets. Moreover, YOLO-v5s [32] proved to be the most promising approach to be run in mobile devices due to its competitive results and its ability to process up to 5.19 frames per second (FPS) in a commercial smartphone.

The remaining of the paper is organized as follows: Section 2 presents and discusses related work. Section 3 introduces PlastOPol, a new dataset for litter detection. Section 4 describes the evaluation protocol adopted for the purpose of the comparison. Section 5 presents and discusses the results and possible limitations of the study. Finally, Section 6 states our conclusions and points out possible research avenues for future work.

## 2. Related Work

This section presents relevant concepts and works related to the object detection problem using deep learning (Section 2.1) and an overview of well-known object detection networks (Section 2.2).

### 2.1. Object Detection Using Deep Learning

Object detection is a computer vision task that involves both localizing and classifying objects within an image, where each detected object is wrapped by a bounding box. Object detection is widely used in several applications, such as self-driving technologies [38], surveillance and security [39] and robotics [40], among others.

As an overview, a neural network for object detection follows the next pipeline; as a first step, looking for regions where objects could be placed, an initial set of bounding boxes is defined via the prior boxes or region proposal techniques. Prior boxes are a predefined set of bounding boxes with different sizes and aspect ratios distributed for the entire image [30,32,41]; on the other hand, region proposal techniques look for regions of interest (RoIs) where the network considers there is a high likelihood of containing an object [28,42,43]. Then, the detector analyzes all the pre-selected regions and makes two predictions for each one of them, (i) coordinates of the predicted bounding boxes and (ii) their corresponding classes. At the end of the process, the network may find multiple bounding boxes surrounding each object. For this, most of the methods use non-maximum suppression (NMS) to discard bounding boxes based on their intersection over union (IoU) and confidence score.

Readers may refer to [20] for more details regarding the state of the art on object detection.

### 2.2. Overview of Deep Learning Approaches for Object Detection

Next, we provide a brief introduction to some of the most popular object detection networks.

- **Faster R-CNN** [28] is based on Fast R-CNN [42]. Faster R-CNN uses a region proposal network (RPN) to propose RoIs. RPN determines where in the image a potential object could be. Additionally, an RoI pooling layer is used to extract fixed-size RoIs that are passed into two fully connected layers, (i) a softmax layer for object classification and (ii) a bounding-box regressor to predict the final location. The pipeline of this method is end-to-end trainable and accelerates the testing time to near-real-time performance.
- **Mask R-CNN** [43] adds a segmentation module in parallel with the existing branches for the class classification and bounding-box regression presented in Faster R-CNN [28]. Mask R-CNN outputs a binary mask at pixel level of the object, bounding-box/class labels and bounding-box regressions.
- **You Only Look Once (YOLO)** [44] is a family of fast object detection networks that can be optimized to achieve real-time performance. YOLO splits the input image into a grid of cells where each cell predicts a bounding box and object classification. There exist several versions of YOLO that use different backbones. Recently, using genetic programming to adjust the set of prior boxes, YOLO-v5 [32] has appeared as the most promising approach for object detection with a fast training stage and outperforming its previous YOLO versions.
- **EfficientDet** [30] is a new scalable and efficient object detection family that uses EfficientNet [45] as backbone. Additionally, a weighted bi-directional feature pyramid network (BiFPN) allows easy and fast multi-scale feature extraction to be performed. Traditional FPN treats features equally, while BiFPN flows in both the top-down and bottom-up directions and adds an additional weight to each input feature, allowing the network to learn the importance of each. Both BiFPN and class/box net layers are repeated multiple times based on different resource constraints.
- **RetinaNet** [41] is a single-stage unified network composed of an FPN on top of an off-the-shelf ResNet and two task-specific subnets. The first task-specific subnet is responsible for predicting the class on the feature maps produced by the backbone, while the second performs bounding box regression. The authors also introduced a novel focal loss function to prevent the vast number of easy negatives from overwhelming the detector during the training.

Table 1 shows some specifications of the approaches presented for object detection; those specifications were taken from their papers or official GitHubs. All the methods could be divided into two well-defined categories, (i) methods that use a predefined set of anchor boxes with different sizes and ratios for prediction (one-stage methods) and (ii) methods which generate region proposals before defining candidate bounding boxes (two-stage methods). Approaches such as RetinaNet [41], EfficientDet [30], and YOLO [44] are one-stage methods, while R-CNN [46], Fast R-CNN [42], Faster R-CNN [28] and Mask R-CNN [43] belong to the two-stage category.

Moreover, as we can see, those methods use well-known backbone structures, such as VGG-16 [21], ResNet [48], and ZF [21], or they propose their own backbone, such as EfficientDet [30] and YOLO [44]. Additionally, these approaches use different inference scales during testing. The inference scale along with their model size give us insights into whether it is possible or not to run them in devices with computational constraints and/or use them for real time applications. In this context, EfficientDet [30] and YOLO-v5 [32] proposed two promising neural network versions for running in mobile devices, EfficientDet-d0 and YOLO-v5s, respectively. EfficientDet-d0 uses EfficientNet-B0 [45] along with a BiFPN as backbone, which produces a model size of 17 Megabytes. In the case of YOLO-v5s, the authors used a small version of their own backbone, building a model of just 15 Megabytes.

## 3. PlastOPol Dataset

There are few datasets dealing with the issue of littering. Proença and Simões [37] presented TACO for litter segmentation with the presence of indoor and outdoor scenes. In the same vein, MJU [33] is another dataset for segmentation; however, unlike TACO, this dataset contains only indoor images with people holding the litter instances in their hands. For its part, Aquatrash [49] is an alternative dataset composed of a subset of images from TACO [37]. Additionally, there are also datasets proposed for sorting recyclable litter, some of them involve detection of waste in industrial plants [35,36] and others for classification in a more controlled set-up with the presence of a single litter instance per image [34]. Except for TACO [37], none the above mentioned datasets present outdoor backgrounds, which is a limitation to build computational solutions for detecting litter in the environment.

Herein, as one of the main contributions of this work, we present a new dataset for litter detection, named “PlastOPol”. The proposal of this dataset has the goal of giving to the computer science and environmental science communities a new set of images with the presence of litter in several types of environments. We hope that PlastOPol serves as basis for the proposal of automatic detection methods which can support the furthering of research on litter in the environment. The images were collected by the Marine Debris Tracker available under an open access Creative Commons Attribution license.

Building the dataset involved the meticulous task of labeling each litter instance in each image. PlastOPol is a one-class labeled dataset, where all the data corresponds to the “litter” class as its super category. This dataset has 2418 images collected by the Marine Debris Tracker with a total of 5300 instances of litter. Each instance is wrapped within a rectangular bounding box represented by four values ($x_{1}$, $y_{1}$, width and height), where ($x_{1}$, $y_{1}$) corresponds to the upper left corner of the bounding box.

**Figure 2:**
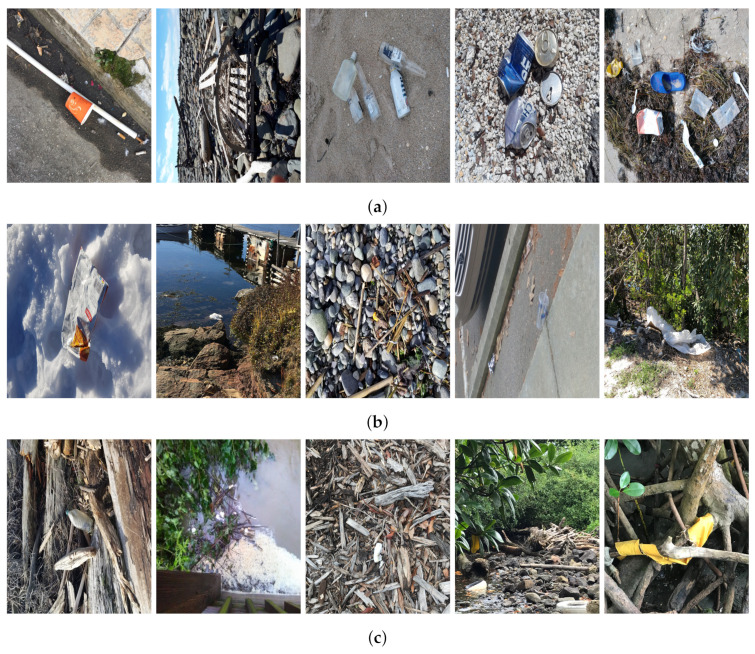

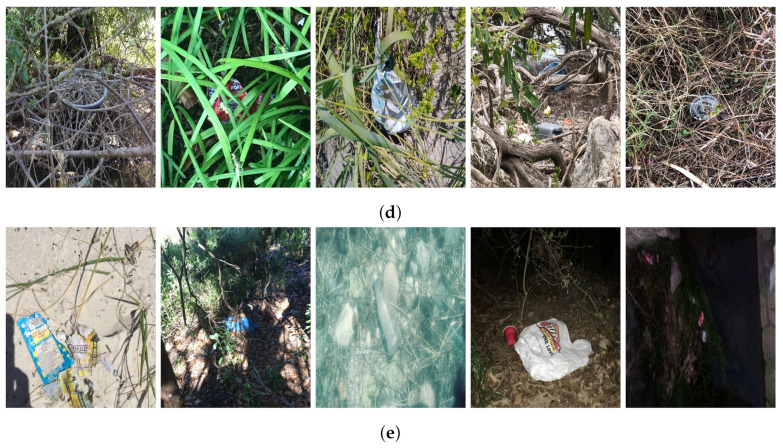
Examples from PlastOPol dataset. (**a**) Types of litter. (**b**) Types of environment. (**c**) Natural Backgrounds. (**d**) Occlusion. (**e**) Lighting.

Based on the bounding boxes areas, 90.98% are considered as large, 8.40% medium and 0.62% small (see Table 2 for details). Additionally, as can be observed in Figure 2, our dataset presents images involving diverse types of environments as background, e.g., water, snow, sand, flint fields, streets, etc.; and various types of litter, including plastic, glass, wood, metal, paper, cloth and rubber, among others. Moreover, PlastOPol contains images with different lighting, occlusion and background conditions, which lead to very challenging detection scenarios. All these elements make the PlastOPol dataset very complete and ideal for training object detection models. Detecting litter from different materials, colors and shapes present in different natural backgrounds with varying illumination conditions increases the chance of confusing or not detecting litter, even more with the presence of small litter instances.

**Figure 3:**
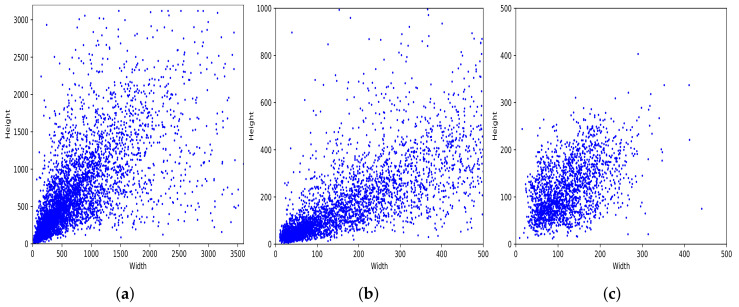
Bounding boxes by size. (**a**) PlastOPol. (**b**) TACO [37]. (**c**) MJU-waste [33].

**Figure 4:**
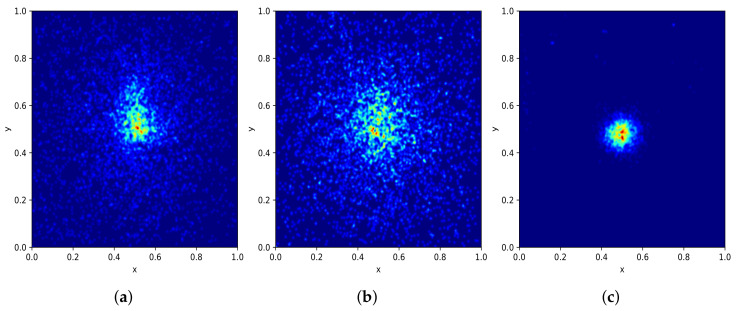
Bounding boxes by location. (**a**) PlastOPol. (**b**) TACO [37]. (**c**) MJU-waste [33].

Figure 3a shows the distribution of bounding boxes based on their width and height. As we can see, our dataset has diverse bounding box sizes, ranging from 36×8 to 4515×2821 pixels. Moreover, it shows a large concentration in the area of 500×500 pixels. Figure 4a shows how the entire set of bounding boxes are located in the images, with most of them being in the center of their respective images; nevertheless, there are also bounding boxes located over the whole image area.

Figure 3 and Figure 4 present some details about the PlastOPol (outdoor scenes), MJU [33] (indoor scenes), and TACO [37] (indoor/outdoor scenes) datasets. PlastOPol contains a wider diversity in terms of the size of bounding boxes, compared with TACO and MJU (see Figure 3). Moreover, PlastOPol (5344×4008 pixels) and TACO (6000×4000 pixels) are composed of images with higher resolution than MJU (640×480 pixels). Regarding the bounding box location, Figure 4 shows the distribution of bounding boxes inside the images. MJU, an indoor dataset created in a controlled set-up, presents almost all their litter instances located in the center of the image, while PlastOPol and TACO have more sparse bounding boxes, covering most of the image area in a realistic and challenging scenario for litter detection.

## 4. Experimental Setup

This section introduces the experimental setup employed in the comparative study, including information about datasets and metrics (Section 4.1), the adopted experimental protocol (Section 4.2) and the procedures for assessing the performance of methods on mobile devices (Section 4.3).

### 4.1. Datasets and Metrics

To evaluate the performance of different deep learning approaches in the context of litter detection, we used two publicly available datasets, (i) our dataset, called “PlastOPol” (the PlastOPol dataset is publicly available under the Creative Commons Attribution license (CC BY 4.0), presented in Section 3; and (ii) TACO [37]. These datasets present litter in several areas, for instance, streets, rivers, beaches, etc.

**Figure 5:**
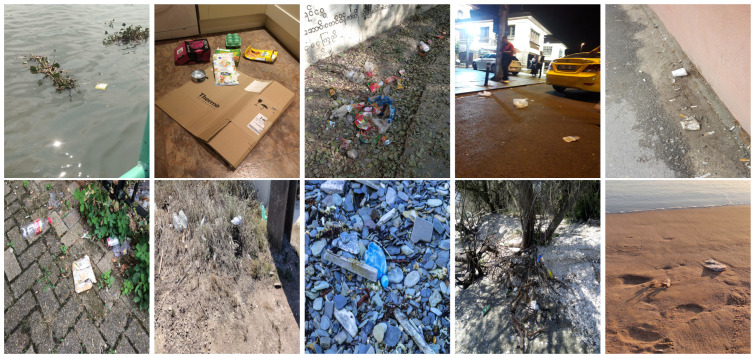
Examples from TACO dataset.

The TACO dataset [37] presents 1500 images involving 4784 annotations, where most of the annotations are considered as large, with images ranging from 842×474 to 6000×4000 pixels. This dataset labeled its images under 60 categories corresponding to 28 super categories; however, an experimental one-category set-up was also proposed by the authors where all the litter instances belong to the “litter” class. TACO is a very challenging dataset due to the presence of bottles, bottle caps, glass, rope, strings, etc. (see Figure 5). Additionally, there is a large number of samples from the cigarette class in the ground truth. This type of litter is covered by bounding boxes with a size less than 64×64 pixels, which makes it a very complex scenario for detection. More details are presented in Table 2.

In order to evaluate the performance of the evaluated deep neural network approaches, we measured (i) the effectiveness in terms of average precision (AP) at an intersection over union (IoU) of 50%, along with the AP@, AR@ and F-measure (F1@); and (ii) the efficiency in terms of model size in Megabytes (MB) and processing time in frames per second (FPS). The COCO metrics (https://cocodataset.org/#detection-eval (accessed on 2 August 2021)) AP@ and AR@ refer to the average precision and average recall, respectively, considering the average of 10 precision-recall pairs computed through changing the IoU value from 50% to 95%, at steps of 5%.

### 4.2. Experimental Protocol

For the experiments, we partitioned the datasets into training, validation, and testing sets. First, we divided the whole dataset in 80% for training and 20% for testing. Then, for training, we used a 5-fold cross validation [50], creating five subsets randomly from the training set, four of them for training and the remaining for validation (this procedure was repeated 5 times). Then, we selected the best model in the validation set from the 5 folds and used it to compute the final results on the test set.

To assess the effectiveness of the evaluated approaches, we considered the same hyper-parameters used by the authors of each approach [28,30,32,41,43]. Table 3 presents details about the hyper-parameters’ values, along with the GitHub repositories for the source codes used in this study (as of 2 July 2021). For the experiments, we used the same code as the GitHub repositories.

### 4.3. Experimental Protocol for Performance Assessment on Mobile Devices

In this study, we also report the results of two tiny neural networks (YOLO-v5s [32] and EfficientDet-d0 [30]) on mobile devices. We selected these neural network approaches because of their compact models and competitive results, 17 MB for EfficientDet-d0 and 15 MB for YOLO-v5s.

These experiments have the goal of giving insights into the performance of these two models in the context of devices with computational constraints such as smartphones and drones, among others. We believe that insights and conclusions found in these experiments will be helpful for other researchers that aim to create real-time applications deployed in computationally restricted devices.

To perform the experiments, we used a commercial smartphone, Motorola Moto-G6, with the following specifications:

- Platform: Android 9;
- CPU architecture: ARMv7;
- Memory: 3 GB;
- Processor: Octa-core—8x1.4 GHz Cortex-A53.

Moreover, in order to evaluate the performance of these approaches and the impact of the input size in devices with computational constraints, we executed experiments using six widely used scales as input: 224×224, 320×320, 384×384, 512×512, 640×640 and 768×768 pixels. To measure the speed of YOLO-v5s [32] and EfficientDet-d0 [30], we used the test set of PlastOPol (484 images) and reported the average time of 5 runs for each scale.

For these experiments, we built a basic Android app; our implementation was based on three GitHub repositories corresponding to PyTorch (https://github.com/pytorch/android-demo-app/tree/master/ObjectDetection (accessed on 2 August 2021)), Tensorflow (https://github.com/tensorflow/examples/tree/master/lite/examples/object_detection/android (accessed on 2 August 2021)) and YOLO-v5 (https://github.com/ultralytics/yolov5/issues/251 (accessed on 2 August 2021)) implementations.

## 5. Results and Discussion

This section presents the results of seven state-of-the-art deep neural networks when applied in the context of litter detection over two datasets, PlastOPol (Section 5.1) and TACO (Section 5.2). We then discuss the effectiveness and efficiency of two compact neural networks (YOLO-v5s [32] and EfficientDet-d0 [30]) on a mobile set-up (Section 5.4). In our comparative study, we considered these tiny deep neural networks with the goal of evaluating the trade-off between effectiveness and efficiency when running these approaches on devices with computational constraints.

### 5.1. Results on PlastOPol

Table 4 presents the effectiveness of the evaluated deep neural networks on the PlastOPol dataset. As we can see, YOLO-v5x [32] reached promising results on this dataset, outperforming the remaining methods with at least 5.0 percentage points in terms of AP50. Moreover, YOLO-v5x was 5.0, 9.6, 11.2, 11.6, 11.7 and 19.9 percentage points more accurate than YOLO-v5s [32], Faster R-CNN [28], Mask R-CNN [43], RetinaNet [41], EfficientDet-d5 [30] and EfficientDet-d0 [30], respectively. Additionally, regarding the tiny approaches, YOLO-v5s surpassed EfficientDet-d0 by 14.9 percentage points.

Table 5 shows some visual results on the PlastOPol dataset. Those results are associated with different types of litter and natural backgrounds. The expected detection (ground truth) is presented in the first column; the confidence threshold for plotting the bounding boxes are the same as the ones used in the papers or GitHub, for instance, 0.4 for EfficientDet, 0.25 for YOLO-v5 and 0.5 for Faster R-CNN, Mask R-CNN and RetinaNet. In the first row of images, we present a case where almost all the methods reached 100% of effectiveness. However, there are a couple of cases corresponding to Faster R-CNN and RetinaNet where false positives were found, i.e., small flints considered as litter. Moreover, the shadow of a bottle caused small errors in the detection of RetinaNet, EfficientDet-d0 and YOLO-v5s, where we can see that these methods covered the bottle with a larger bounding box wrapping it along with its shadow. In the second image, showing litter in an urban context, all the methods missed one instance of litter. Additionally, RetinaNet had some false positives, even considering a car as litter.

The third row presents scenarios with challenging natural backgrounds. In this case, almost all the methods produced erroneous detection results, except for EfficientDet-d5. In the fourth row, the image presents several instances of litter with different types of materials, different sizes, colors and shapes. In that image, Faster R-CNN and RetinaNet had a lot of false positives, EfficientDet-d0 missed some detections and there was one occluded litter item located almost at the center of the image that was not detected for any of the approaches.

Finally, the last two rows present results associated with special detection scenarios. The penultimate row shows a plastic bag as litter, but we can see that most of the methods detected the recycling bins as litter as well. This scenario illustrates how the definition of litter depends on the context of the image and the place where it was taken, i.e., some artifacts, such as the recycling bins, could be considered as litter if they were in another area, such as a beach, a forest, a water course etc. The last row presents an image with the presence of cigarette butts, which are small litter instances. As we can observe, none of the evaluated methods could detect all of the items and some (RetinaNet and Faster R-CNN) led to more detection errors than others (Mask R-CNN, EfficientDet and YOLO-v5). Detecting small items, in a crowded image and with a noisy background, is one of the hurdles for the evaluated methods.

### 5.2. Results on TACO

We also measured the effectiveness of the evaluated approaches on TACO [37].This dataset presents overlapping litter and small litter instances which make the detection more challenging. On this dataset, in terms of AP50, YOLO-v5x [32] outperformed the other methods with at least 8.6 percentage points, with an AP50 of 63.3, showing its superiority on this dataset over the other methods (see Table 6). As for YOLO-v5s [32] and Mask R-CNN [43], the second and third best performing methods, YOLO-v5x reduced the detection errors of these approaches by 18.98% and 23.06%, respectively. YOLO-v5x and YOLO-v5s achieved better results in the detection of medium and large objects. Nonetheless, all the methods had issues detecting small objects. Additionally, among the approaches with smaller model size, YOLO-v5s obtained better results than EfficientDet-d0 with a considerable AP50 difference of 22 percentage points.

In Table 7, we present some visual results to illustrate the performance of the methods in challenging scenarios for detection on TACO [37]. As mentioned before, TACO annotations involve several cases with bounding boxes inside one another, which creates a more complex scenario. To plot the bounding boxes, we used the same threshold value of confidence scores suggested in the original papers or implementations, i.e., 0.4 for EfficientDet [30], 0.25 for YOLO-v5 [32] and 0.5 for Faster R-CNN [28], Mask R-CNN [43], and RetinaNet [41].

In the first row, we can see an apparently “simple” scenario, with the presence of three instances of litter. According to the TACO annotations, the cup is divided in two litter instances, i.e., the cup itself and its lid, which makes it a very challenging scenario for litter detection. In this case, all the methods detected the cup along with its lid as one instance. Moreover, Faster R-CNN and YOLO-v5x had some overlap bounding boxes in one of the litter instances. In the second image, another “simple” example is presented where only RetinaNet [41], EfficientDet-d0 [45] and EfficientDet-d5 [45] could detect the only instance correctly; the remaining methods generated overlapping bounding boxes. Similar behavior was observed in the third image, in which only EfficientDet-d5 [45] detected correctly all the litter instances without false positives. Moreover, as we can see, Faster R-CNN [28], Mask R-CNN [43] and RetinaNet [41] produced erroneous overlapping bounding boxes when different instances of litter were placed close together.

The last three images present challenging natural backgrounds where none of the evaluated methods could detect all the litter instances. In the fourth image, only Faster R-CNN [28] and RetinaNet [41] detected the three instances of litter, but both with a false positive. In the penultimate row, RetinaNet [41], EfficientDet-d5 [45], and YOLO-v5x [44] performed best; nevertheless, all of them had errors. Finally, the last image presents a very complex scenario, including very small objects (labeled as A, B, C and D in the ground truth image). None of the methods could detect those objects, possibly due to their sizes combined with the complex natural background.

### 5.3. Effectiveness vs. Efficiency

When it comes to the trade-off between effectiveness and efficiency, we took into account the results on both datasets, the model sizes and processing time to compare the networks. In the case of processing time, we considered only the network time process. The pre- and post-processing stages are not reported in the next results. On an NVIDIA GeForce Titan X with 12 GB, as expected, the approaches with light models were the fastest. YOLO-v5s [32] and EfficientDet-d0 [30] were capable of processing up to 73.75 FPS and 59.35 FPS, respectively. Considering the best methods that produce “heavy” models, YOLO-v5x was 2.93× and 3.33× faster than Faster R-CNN [28] and Mask R-CNN [43], respectively.

**Figure 6:**
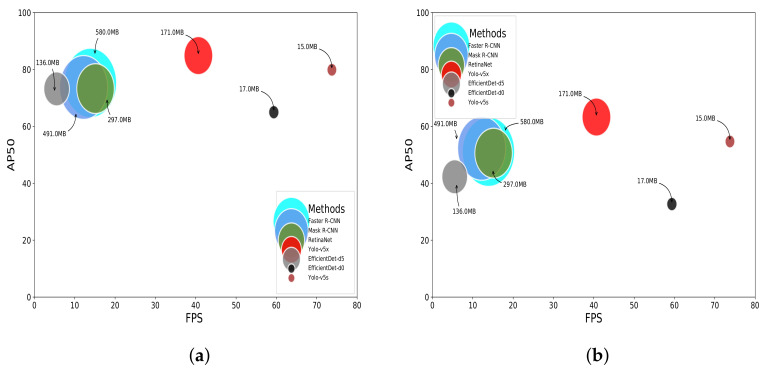
Efficiency (GPU) vs. effectiveness vs. model size. (**a**) PlastOPol. (**b**) TACO.

Figure 6 clarifies the differences between the methods in terms of trade-off between effectiveness (AP50) and efficiency, considering model size and FPS. In this set-up, clearly, YOLO-v5s [32] offered the best trade-off, with an AP50 of 79.9 and 54.7 for the PlastOPol and TACO datasets, respectively. Moreover, this network presents a model size of 15 MB and a processing time of at least 14.4 FPS more than its counterparts and being capable of processing up to 73.75 FPS.

EfficientDet-d0 [30] also presented promising results, especially considering its model size and FPS. However, this network presented a considerable miss detection rate, which could be improved without impacting its current efficiency too much. On the other hand, YOLO-v5x [32] is a very powerful approach that obtained the best AP50 in both datasets with a model size of 171 MB and 40.65 FPS processing. Nevertheless, the model size of YOLO-v5x limits this network from running in devices with computational constraints for real-time applications.

### 5.4. Efficiency on Mobile Devices

This section presents some experiments to evaluate the performance of YOLO-v5s [32] and EfficientDet-d0 [30] on mobile devices. These two approaches are presented in their respective papers as compact versions of their original proposals focusing on the trade-off between effectiveness and efficiency. First, we prepared both models to run on a mobile environment according to their official GitHubs (https://github.com/google/automl/tree/master/efficientdet (accessed on 2 August 2021); https://github.com/ultralytics/yolov5/pull/1127 (accessed on 2 August 2021)), obtaining their corresponding TFlite models, 17 Megabytes for EfficientDet-d0 and 28 Megabytes for YOLO-v5s.

For this purpose, we used a Motorola Moto-G6 as a commercial smartphone to run our experiments. Details about the smartphone, app and protocol are described in Section 4.3. For comparative purposes, the experiments considered only the neural network processing time and neither pre- nor post-processing phases were taken into account.

**Figure 7:**
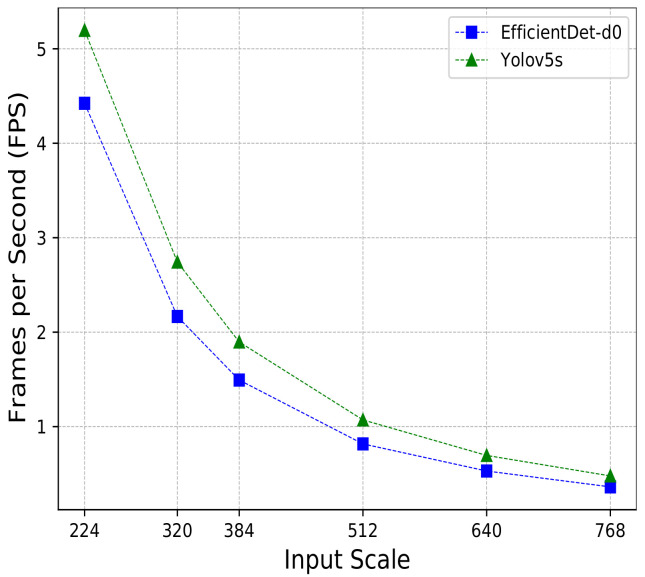
Efficiency on Motorola Moto-G6.

Figure 7 shows the results using six commonly used input sizes, 224×224, 320×320, 384×384, 512×512, 640×640 and 768×768. Both neural networks had a similar behavior, i.e., increasing the size of the input impacted directly on the processing time. Using an input size of 224×224, the methods were able to process up to 5.19 FPS for YOLO-v5s and 4.42 for EfficientDet-d0. In all the experiments, YOLO-v5s was faster than EfficientDet-d0, with a speedup ranging from 1.17× to 1.30× across the different scales. Furthermore, the figure shows that, despite the use of compact models, the FPS rate dropped dramatically when larger images were processed. The FPS rate decreased from 5.19 FPS (224×224) to 0.48 FPS (768×768) in the case of YOLO-v5s and decreased from 4.42 FPS (224×224) to 0.36 FPS (768×768) when EfficientDet-d0 was considered.

## 6. Conclusions

One of the most pressing environmental challenges of our time is how to mitigate the impact of littering in the environment. In this work, we present a new dataset, called “PlastOPol,” which contains images of various types and sizes of litter with different real-world backgrounds (streets, forest and beaches, among others) and annotations locating the litter within the images. Our goal is to foster the creation of more effective and efficient machine learning solutions for litter detection and classification with the ultimate aim of automating the process.

We performed a comparative study involving state-of-the-art approaches for object detection using the PlastOPol and TACO datasets. The overall performance of the evaluated methods was similar for both datasets. We also observed that the YOLO-v5-based object detectors performed best. On PlastOPol, YOLO-v5x reached an AP50 of 84.9, followed by YOLO-v5s with 79.9. On TACO, the AP50 observed was 63.3 and 54.7 for YOLO-v5x and YOLO-v5s, respectively. There were challenging backgrounds where several methods had false positives, confusing wood with litter, for instance. Moreover, all the methods had problems detecting small objects, such as cigarette butts.

We also evaluated the performance of the methods. On GPU, YOLO-v5s and EfficientDet-d0 were the fastest approaches, processing up to 73.75 and 59.35 FPS, respectively, and YOLO-v5x was capable of processing at 40.65 FPS. As for the remaining methods, YOLO-v5s was 4.87×, 5.31×, 6.05× and 13.38× faster than RetinaNet, Faster R-CNN, Mask R-CNN and EfficientDet-d5, respectively.

In the last years, most of the approaches proposed to improve object detection have been using very deep neural network architectures, which increases their model size, making it, in practice, almost impossible to use in devices with computational restrictions and real-time applications. Unlike other areas, the optimization of automatic litter detection involves searching for litter, even in areas of difficult access, using devices, such as drones or mobile devices. For this reason, we also compared the performance of EfficientDet-d0 and YOLO-v5s, which are two light models, in a real mobile set-up using a commercial smartphone. YOLO-v5s was the fastest one in all the experiments. With an image input size of 224×224, YOLO-v5s was capable of processing at 5.19 FPS, being 1.17× faster than EfficientDet-d0. Moreover, increasing the input size reduced the efficiency of the models drastically. With an input size of 768×768 pixels, YOLO-v5s and EfficientDet-d0 processed at just 0.48 and 0.36 FPS, respectively.

In summary, we demonstrated how well state-of-the-art object detection methods performed on two challenging datasets for litter detection, as well as their strengths and weaknesses. All methods had limitations when presented with difficult scenarios, with complex natural background and very small litter instances. Future research efforts should focus on improving the litter detection of the state-of-the-art methods using fusion strategies [51]. We also plan to continue investigating how tiny deep neural networks can run on devices with computational constraints, also considering well-known lightweight object detection methods, such as MobileNet [52], ShuffleNet [53] and Pelee [54], among others. Moreover, having few samples of small instances impacts the detection rate in these types of instances, as the detectors do not have enough information to capture a better description of this type of litter during training. Therefore, there is a greater probability of not detecting these instances during testing. For this reason, the extension of our dataset using smart data augmentation strategies, especially for very small litter instances, will be investigated.

Finally, PlastOPol does not contain annotations related to the types of litters (e.g., plastic, paper and metal, among others) found within images. We plan to provide such multiclass annotations in the future, assess the performance of the state-of-the-art methods and also propose a new approach to detecting litter in a multiclass set-up. Furthermore, with the proposal of a multiclass dataset, we intend to analyze the balance of instances per class and its impact on efficiency. In addition, it is also important to evaluate the impact of the dataset size by evaluating the effectiveness of the methods on a subset of images with the goal of prioritizing the dataset distribution rather than its size.

## Figures and Tables

**Table 1:** Comparison of deep learning approaches for object detection.

CNN Methods	One-Stage	Two-Stage	Backbone	Inference Scale	Model Size (MB)
Faster R-CNN		✓	VGG-16 [21]	Shorter side 600	580.0
		✓	ZF [21]	Shorter side 600	242.5
Mask R-CNN		✓	ResNet-101-FPN	Shorter side 600	491.0
RetinaNet	✓		ResNet-50-FPN	500×500	297.0
			ResNet-101-FPN	500×500	442.0
			ResNet-101-FPN	800×800	442.0
EfficientDet	✓		EfficientNet-B0-BiFPN	512×512	17.0
	✓		EfficientNet-B1-BiFPN	640×640	28.0
	✓		EfficientNet-B2-BiFPN	768×768	35.0
	✓		EfficientNet-B3-BiFPN	896×896	51.0
	✓		EfficientNet-B4-BiFPN	1024×1024	85.0
	✓		EfficientNet-B5-BiFPN	1280×1280	136.0
	✓		EfficientNet-B6-BiFPN	1280×1280	206.0
	✓		EfficientNet-B7-BiFPN	1536×1536	208.0
YOLO	✓		Own	448×448	–
	✓		VGG-16 [21]	448×448	–
YOLOv2	✓		Own-Darknet-19	480×480	194.0
	✓		Own-Darknet-19	544×544	194.0
YOLOv3	✓		Own-Darknet-53	320×320	237.0
	✓		Own-Darknet-53	416×416	237.0
	✓		Own-Darknet-53	608×608	237.0
YOLOv4	✓		CSPDarknet53 [47]	416×416	246.0
	✓		CSPDarknet53 [47]	512×512	246.0
	✓		CSPDarknet53 [47]	608×608	246.0
YOLOv5	✓		Own-5s	640×640	15.0
	✓		Own-5m	640×640	42.0
	✓		Own-5l	640×640	92.0
	✓		Own-5x	640×640	171.0

**Table 2:** Datasets employed in the comparative study.

	#	# Bounding Boxes by Area			#
Dataset	Images	Small ${}^{1}$	Medium ${}^{2}$	Large ${}^{3}$	Annotations
TACO [37]	1500	384	1305	3095	4784
PlastOpol	2418	33	445	4822	5300

^1^ Small→area ≤ 32^2^. ^2^ Medium→32^2^ < area ≤ 96^2^. ^3^ Large→area > 96^2^.

**Table 3:** Training protocol values.

	Hyper-Parameters						
Method	Input Size	Lr	Epochs	Batch Size	${\mathrm{Lr}}_{\mathrm{decay}}$	Post-Processing	Confidence Threshold
EfficientDet-d0 ${}^{1}$	512×512	0.08	300	48	200, 250	Soft-NMS	0.4
EfficientDet-d5 ${}^{1}$	1280×1280	0.08	300	12	200, 250	Soft-NMS	0.4
Faster R-CNN ${}^{2}$	800×800	0.0001	300	8	243	NMS	0.5
Mask R-CNN ${}^{2}$	800×800	0.0001	300	8	243	NMS	0.5
RetinaNet ${}^{2}$	800×800	0.0001	300	8	243	NMS	0.5
YOLO-v5x ${}^{3}$	640×640	0.0032	100	12	–	NMS	0.001
YOLO-v5s ${}^{3}$	640×640	0.0032	100	12	–	NMS	0.001

^1^https://github.com/google/automl/tree/master/efficientdet (accessed on 2 July 2021). ^2^ https://github.com/facebookresearch/detectron2 (accessed on 2 July 2021). ^3^ https://github.com/ultralytics/yolov5 (accessed on 2 July 2021). **Lr**—learning rate. **Lr_decay_**—epochs in which the learning rate is decayed.

**Table 4:** Litter detection results on PlastOPol (best results appear in bold).

Methods	AP50	AP@	AR@	F1@
RetinaNet [41]	73.3	47.2	56.4	51.4
Faster R-CNN [28]	75.3	49.6	57.0	53.0
Mask R-CNN [43]	73.7	50.2	57.2	53.5
EfficientDet-d0 [30]	65.0	51.1	56.1	53.5
EfficientDet-d5 [30]	73.2	69.1	66.4	67.7
YOLO-v5s [32]	79.9	62.4	76.9	68.9
**YOLO-v5x [32]**	**84.9**	**71.1**	**82.1**	**76.2**

**Table 5:** PlastOPol visual results.

Ground Truth	Faster R-CNN	Mask R-CNN	RetinaNet	EfficientDet-d0	EfficientDet-d5	YOLO-v5s	YOLO-v5x
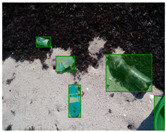	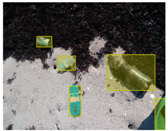	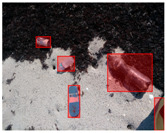	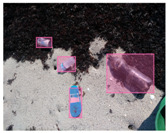	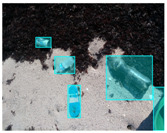	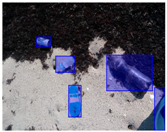	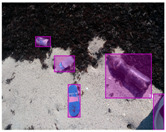	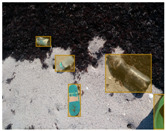
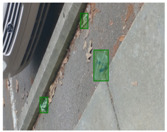	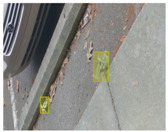	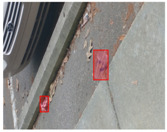	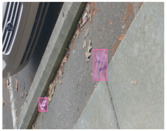	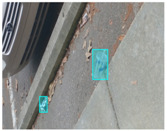	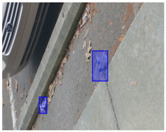	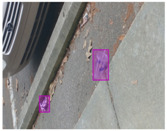	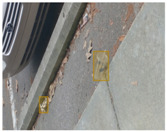
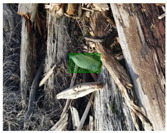	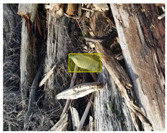	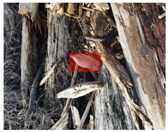	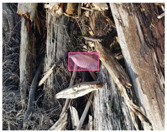	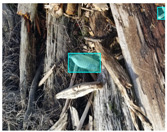	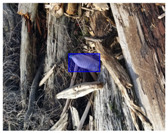	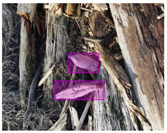	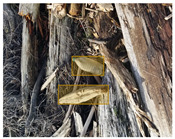
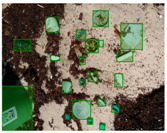	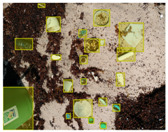	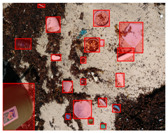	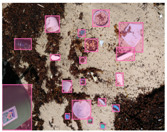	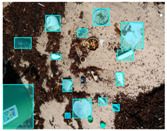	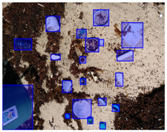	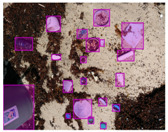	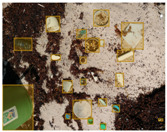
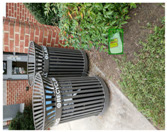	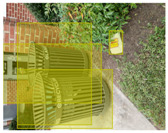	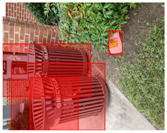	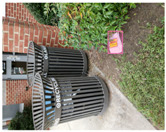	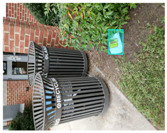	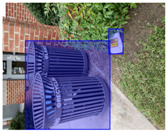	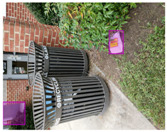	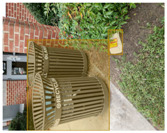
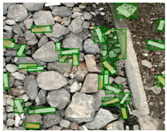	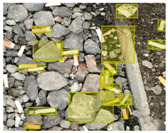	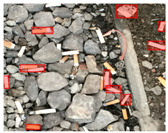	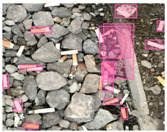	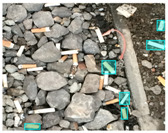	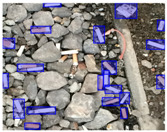	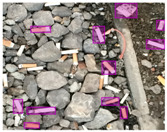	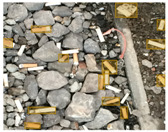

**Table 6:** Litter detection results on TACO (best results appear in bold).

Methods	AP50	AP@	AR@	F1@
RetinaNet [41]	50.6	26.7	37.1	31.1
Faster R-CNN [28]	51.1	28.1	36.9	31.9
Mask R-CNN [43]	52.3	29.2	38.6	33.2
EfficientDet-d0 [30]	32.7	23.8	28.4	25.9
EfficientDet-d5 [30]	42.3	35.2	40.3	37.6
YOLO-v5s [32]	54.7	38.8	58.1	46.5
**YOLO-v5x [32]**	**63.3**	**48.4**	**66.4**	**56.0**

**Table 7:** TACO visual results.

Ground Truth	Faster R-CNN	Mask R-CNN	RetinaNet	EfficientDet-d0	EfficientDet-d5	YOLO-v5s	YOLO-v5x
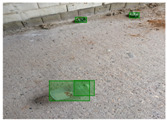	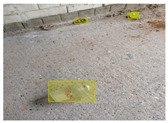	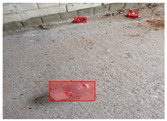	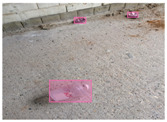	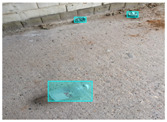	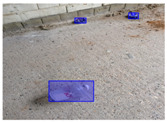	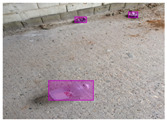	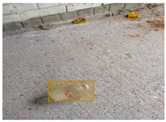
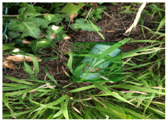	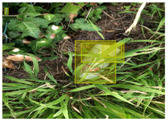	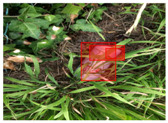	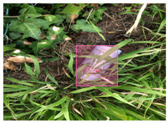	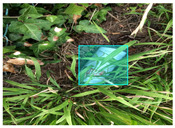	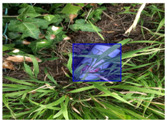	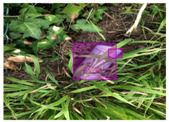	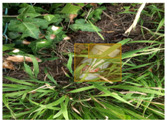
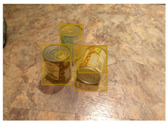	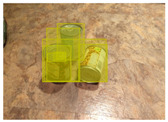	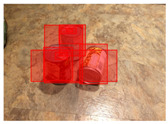	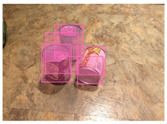	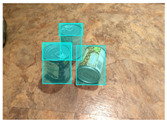	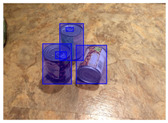	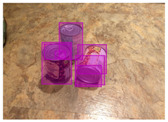	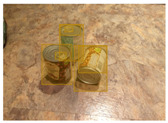
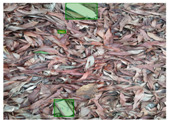	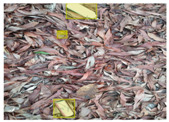	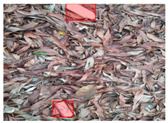	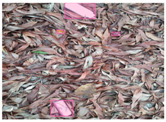	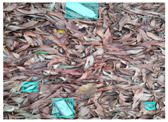	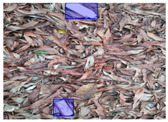	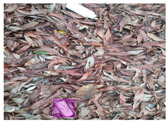	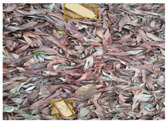
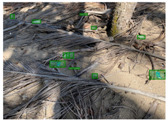	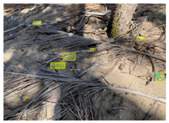	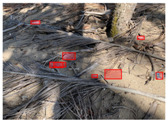	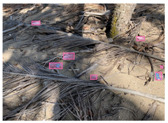	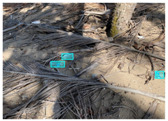	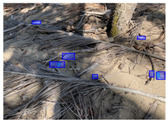	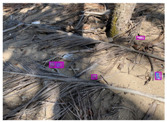	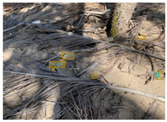
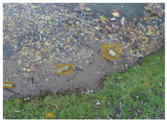	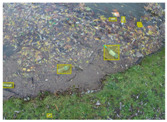	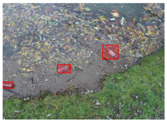	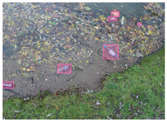	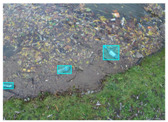	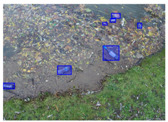	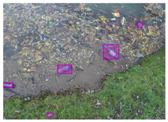	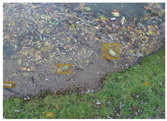

## Data Availability

PlastOpol dataset is available at https://zenodo.org/record/5829156 (accessed on 1 January 2022). PlastOpol is publicly available under the Creative Commons Attribution license (CC BY 4.0).
